# Two Rooted Maxillary Lateral Incisor: A Case Report

**Published:** 2012-10-13

**Authors:** Mandeep Singh Matta

**Affiliations:** Rayat Bahra DentalCollege, Mohali, Punjab, India

**Keywords:** Tooth Root, Incisor, Rare Anomaly

## Abstract

An accurate diagnosis of the morphology of the root canal system is a pre-requisite for successful root canal treatment. Frequently, root canals are left untreated because the clinicians fail to identify their presence, particularly in teeth that have anatomical variations or additional root canals. In this report a maxillary lateral incisor with two roots has been described.

## Introduction

Many anatomical studies have declared that maxillary incisors always have a single root, while variations in the number of lateral canals and/or position of apical foramen are reported [1][2][3][4][5]. As indicated in the studies of canal anatomy, multiple canals and roots in maxillary incisors are rare [6][7][8][9][10][11][12].

A brief literature review revealed 11 cases reporting maxillary lateral incisors with two roots [5][9][10][11][12][13][14][15][16][17][18], 7 cases presenting maxillary central incisors with two roots [19][20][21][22][23][24][25] and 4 cases with two root canals in maxillary incisors [26][27][28][29].

Neville et al. used the term supernumerary roots when describing the presence of additional roots on a tooth compared with the classical description in dental anatomy. The most frequently affected teeth are the permanent molars (especially the third molar) from either arch and mandibular cuspids and premolars [30].

Periapical radiography is an essential tool in diagnosing internal anatomy of a tooth. The use of shift cone angle radiographic technique and parallel angle radiograph to identify superimposed roots and overlapping and unidentified canals has been advocated. In this case report, a rare case of maxillary lateral incisor with two roots is described.

## Case report

**Figure 1:**
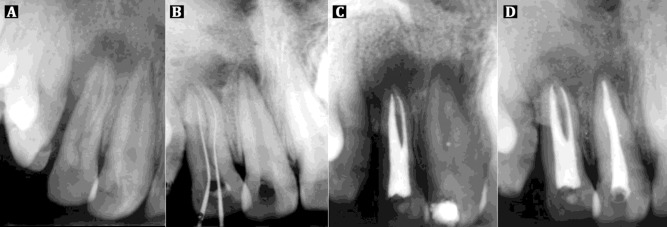
A) Preoperative periapical radiograph; B) Working length periapical radiograph; C) Post obturation periapical radiograph; D) Follow-up radiograph after 1 year

A 20 year-old male was referred to the clinic for root canal treatment of the maxillary right lateral incisor (#7). The medical history was found to be non-contributory. Patient’s chief complaint was pain in relation to the upper right anterior teeth region. Clinical examination showed a pit over the cingulum region. Tooth also had grade I mobility and was tender both on palpation and percussion. No sinus tract or fistula was found. Radiographic examination using bisecting angle technique revealed widening of periodontal ligament space and well-defined periapical radiolucency around the lateral incisor as well as the central incisor (Figure 1A).

Based on the clinical and radiographic evidences, the tooth was diagnosed as having chronic apical periodontitis with an acute episode.

The tooth was isolated with rubber dam (The Hygenic Corporation, Akron, OH, USA) and anaesthetized with 1:200000 solution of Xylocaine with adrenaline (Septodont India Pvt. Ltd, New Delhi, India) using an aspirating syringe. The access was created initially with a no. 2 round bur and then the cavity was enlarged to a triangular outline with round end tapered bur TR 13 (Bur range from Mani. Inc, Togichi, Japan). A small standardized #06 K-file (Dentsply, Maillefer, Ballaigues, Switzerland) was used to negotiate each of the two separate root canals. Conventional working length radiograph was taken (Figure 1B). The mesial orifice was enlarged with Gates Glidden burs (Mani Inc., Togichi, Japan), while the distal canal was wide and therefore the apex was easily negotiated. The two canals were thoroughly instrumented and shaped using stainless steel K files with step back technique to size #25 (apical preparation) for mesial canal and size #50 for the distal canal.

Sodium hypochlorite 3% (Vishal Dentocare Pvt. Ltd., Ahmedabad, India) alternated with 17% EDTA gel (Glyde, Dentsply, Maillefer, Switzerland) was used for irrigation to facilitate shaping. The canals were then dried with sterile paper points (Dentsply, Maillefer, Ballaigues, Switzerland), filled with mixed calcium hydroxide (Neelkanth Healthcare Pvt. Ltd., Jodhpur, Rajasthan, India) and 0.2% chlorhexidine (Safe Plus, Neelkanth Healthcare Pvt. Ltd., Jodhpur, Rajasthan, India) slurry; the coronal cavity was sealed with Cavit G (3M ESPE, 3M Center St. Paul, MN). The access opening for the adjoining central incisor was also performed on the same day with calcium hydroxide and chlorhexidine dressing. A week later, tooth was non-tender to percussion and the canals were dry. The canals were obturated with laterally condensed gutta-percha (Dentsply, Maillefer, Ballaigues, Switzerland). Resin based AH Plus (Dentsply, Maillefer, Ballaigues, Switzerland) sealer was used for obturation. The central incisor showed exudate, so it was treated again with calcium hydroxide/chlorhexidine dressing.

A final radiograph for the lateral incisor (Figure 1C) was then taken and provisional restorations were provided for both the incisors. Patient was recalled after a week and permanent restoration of Type II Glass Ionomer Cement (GC America, Alsip, IL, United States) was placed in the lateral incisor and central incisor was obturated with laterally condensed gutta-percha and Resin based AH Plus sealer.

After 1 year on follow-up visit, the patient reported complete alleviation of symptoms along with reduction in mobility. The recall radiograph showed resolution of the periapical pathology (Figure 1D).

## Discussion

When a maxillary incisor presents with two roots or two root canals, conditions such as fusion, gemination, dens in dente, palatogingival or distolingual groove and some variation in the normal development of Hertwig's epithelial root sheath must be considered [18][27][30].

Gemination is an anomaly in which the tooth germ divides during the development of the tooth, resulting in the formation of a double crown with single root, and in the case of fusion, the crown of two separate tooth buds fuse during development resulting in a bifid crown with two root canals in one root. In this case clinical examination as well as the pre-treatment radiographs revealed a crown of normal size and shape when compared with the contra lateral side. Therefore a diagnoses of fusion (single larger crown) or germination (fused or joined crown) can be disregarded [29][30].

There are few reports of maxillary lateral incisor with dens in dente and dens invaginatus showing two roots [5][6][8][9][10][11]. In the present case the pretreatment radiograph showed no evidence of enamel or dentinal invagination, thus making dens in dente or dens invagination unlikely causative factors.

Another developmental anomaly, which may appear similar to this case radiographically, is palatogingival or distolingual groove, but clinical examination ruled it out [18][27].

According to Bhasker [30] normal root development occurs when Hertwig’s root sheath is horizontally bent at the cementoenamel junction to narrow the cervical opening of the tooth germ. In this case report the clinical crown has normal shape (identical to left maxillary central incisor), it seems that during the epithelial diaphragm formation some incident caused the development of a horizontal flap of the Hertwig’s epithelial root sheath, and then the horizontal flap fused, resulted in the formation of a second root. The slight depression which is present at the mesiobuccal cervical portion of this tooth seems to be the bifurcation area [31]. Undetected mesial horn exposure of the pulp can be considered the trigger for the partial necrosis of the pulpal tissue, which in turn induced the formation of periapical pathosis associated with mesial supernumerary root.
